# Influence of Curing Time on the Microbiological Behavior of Bulk-Fill Nanohybrid Resin Composites

**DOI:** 10.3390/polym13172948

**Published:** 2021-08-31

**Authors:** Andrei C. Ionescu, Allegra Comba, Eugenio Brambilla, Nicoleta Ilie, Lorenzo Breschi, Milena Cadenaro, Nicola Scotti

**Affiliations:** 1 Oral Microbiology and Biomaterials Laboratory, Department of Biomedical, Surgical and Dental Sciences, Università degli Studi di Milano, via Pascal, 36, 20133 Milano, Italy; eugenio.brambilla@unimi.it; 2 Department of Surgical Sciences, University of Turin, via Nizza, 230, 10126 Torino, Italy; alle_comba@yahoo.it (A.C.); nicola.scotti@unito.it (N.S.); 3 Department of Biomedical and Neuromotor Sciences, DIBINEM, University of Bologna, Alma Mater Studiorum, Via San Vitale 59, 40125 Bologna, Italy; lorenzo.breschi@unibo.it; 4 Department of Conservative Dentistry and Periodontology, University Hospital, Ludwig-Maximilians-University, Goethestr. 70, 80336 Munich, Germany; nilie@dent.med.uni-muenchen.de; 5 Department of Medical Sciences, University of Trieste, 34125 Trieste, Italy; mcadenaro@units.it; 6 Institute for Maternal and Child Health IRCCS “Burlo Garofolo”, via dell’Istria 65/1, 34137 Trieste, Italy

**Keywords:** bulk fill, composite resin, restorative materials, surface characterization, energy-dispersive X-ray spectroscopy, scanning electron microscopy, microbial adherence, bioreactor, bacterial biofilm, *Streptococcus mutans*

## Abstract

This in vitro study aimed to evaluate the influence of curing time on surface characteristics and microbiological behavior of three bulk-fill resin-based composites (RBCs). Materials were light-cured for either 10 s or 80 s, then finished using a standard clinical procedure. They were characterized by surface morphology (SEM), surface elemental composition (EDS), surface roughness (SR), and surface free energy (SFE). Microbiological behavior was assessed as *S. mutans* adherence (2 h) and biofilm formation (24 h) using a continuous-flow bioreactor. Statistical analysis included a two-way ANOVA and Tukey’s test (*p* < 0.05). Materials differed substantially as filler shape, dimension, elemental composition and resin matrix composition. Significant differences between materials were found for SR, SFE, and microbiological behavior. Such differences were less pronounced or disappeared after prolonged photocuring. The latter yielded significantly lower adherence and biofilm formation on all tested materials, similar to conventional RBCs. Improved photoinitiators and UDMA-based resin matrix composition may explain these results. No correlation between surface characteristics and microbiological behavior can explain the similar microbiological behavior of bulk-fill materials after prolonged photocuring. This different performance of bulk-fill materials compared with conventional RBCs, where surface characteristics, especially surface chemistry, influence microbiological behavior, may have important implications for secondary caries occurrence and restoration longevity.

## 1. Introduction

Bulk-fill resin-based composites (RBCs) have been introduced in the last decade to simplify restoration procedures, allowing for a higher depth of cure of a single increment (they can be placed in ≥4 mm thick bulks instead of the conventional incremental placement of 2 mm-thick layers) [1,2,3,4,5,6]. This goal was reached by modifying the composition of an RBC, for instance, by lowering the filler content by volume in low-viscosity bulk fills and increasing the dimensions of filler particles (>20 microns); thus, decreasing their specific surface areas. It was shown that these modifications improved light transmission, while lower filler content decreases hardness with no change in the suggested curing time [5,6].

Improving the depth of cure of these polymeric materials was obtained by allowing more light generated by the curing unit to penetrate. This improvement was also made possible by equalizing refractive indices of resin monomers and fillers in the unpolymerized material and incorporating highly reactive photoinitiators. Furthermore, the polymerization shrinkage and the consequent stress on the interfaces were reduced using high-molecular-weight monomers to improve marginal adaptation [7,8]. Bulk-fill RBCs are, in fact, a family of materials that differ from conventional RBCs in many ways, ranging from strategies adopted to enhance its translucency, such as a reduction in the content of the pigment and the use of larger filler particles, to significant changes in the chemical composition such as the use of high-molecular-weight monomers and stress-relieving monomers [5,9,10,11].

Additionally, their degree of conversion, that is, the amount of monomers that react forming the polymeric chains may be enhanced by adding highly reactive photoinitiators [3,5,12]. As shown by several studies, both characteristics can significantly impact the biological behavior of the polymeric material, especially biofilm formation [13,14,15]. In fact, the clinical behavior of bulk-fill materials seems to be just similar to conventional RBCs [1,5], and the influence of the dental healthcare provider’s experience on the clinical outcome when placing polymer-based restorations may be non-neglectable [16].

An essential factor in caries development is bacterial colonization, which leads to biofilm formation on all oral surfaces, both natural and artificial. Full-grown biofilm consists of several bacterial species forming an ecological unit, which are not necessarily involved in dental caries; depending on its composition, a biofilm can be detrimental, neutral, or even beneficial [17,18,19]. In particular, cariogenic biofilm shows a high prevalence of acidogenic and acid-resistant species, such as streptococci and lactobacilli. The prevalence of cariogenic microorganisms in the biofilm community is an imbalance representing the first stage of both the primary and secondary caries formation [17,20]. Furthermore, mutans streptococci adherence and colonization of the surfaces of restorative materials are essential elements in secondary caries development [19]. From this point of view, the surface characteristics of restorative materials are particularly interesting as they influence how materials interact with the oral environment throughout their lifespan, posing critical challenges to their longevity [21].

Lastly, it was shown that light-curing characteristics significantly influence the degree of conversion of an RBC [22,23], affecting biofilm formation [24]. However, literature data on the influence of surface properties on microbial colonization of bulk-fill RBCs is limited and not systematically addressed [13,25,26,27,28]. Therefore, this in vitro study aimed to evaluate the adherence and biofilm formation by *Streptococcus mutans* on the surfaces of three bulk-fill RBCs. The null hypotheses were that (i) there is no difference in bacterial adherence or biofilm formation between the tested bulk-fill materials and (ii) there is no influence of the curing time on the microbiological behavior of the tested bulk-fill materials.

## 2. Materials and Methods

### 2.1. Specimen Preparation

Three different bulk-fill materials were tested, differing in type (one low-viscosity and two high-viscosity) and chemical composition. A flowable RBC with a higher viscosity than a conventional flowable was chosen as a control, having a similar resin/filler ratio to the tested bulk-fill materials (Table 1).

A total of 48 disks with 6 mm diameter and 2 mm thickness were prepared for each tested RBC [28,29]. For the preparation of each disk, a PTFE template was employed. The template was separated from the bench with a glass plate; another glass plate was placed on top to prevent the formation of an oxygen-inhibited layer. Disks from each group were randomly divided into two sub-groups (*n* = 24 each) and light-cured (Celalux 2, Voco, Cuxhaven, Germany) at 1000 mW/cm^2^ for 10 s or 80 s, respectively. Once cured, the disks were stored at 37 °C for 24 h. After that, surfaces were polished with sandpapers of increasing grit size until reaching #1200 (SiC waterproof abrasive sheet, 3M, St Paul, MN, USA). The specimens were then sonicated for two hours in distilled water to remove debris from the finishing procedures and stored separately under light-proof conditions at 37 °C in artificial saliva for six days to minimize the impact of residual monomer leakage or initial fluoride burst on the bacterial cells’ viability. The artificial saliva used in the present study reproduced the average electrolytic composition of whole human saliva and was prepared by mixing 100 mL of 150 mM KHCO_3_, 100 mL of 100 mM NaCl, 100 mL of 25 mM K_2_HPO_4_,100 mL of 24 mM Na_2_HPO_4_, 100 mL of 15 mM CaCl_2_, 100 mL of 1.5 mM MgCl_2_, and 6 mL of 25 mM citric acid. The volume was prepared up to 1 L, and the pH was adjusted to 7.0 by pipetting NaOH 4 M or HCl 4 M solutions under vigorous stirring [29].

### 2.2. Analysis of Specimen Morphology and Elemental Surface Distribution by Scanning Electron Microscopy (Sem) and Energy-Dispersive X-ray Spectroscopy (Eds)

SEM/EDS analyses were performed on four specimens for each group using a tabletop scanning electron microscope (TM4000Plus, Hitachi, Schaumburg, IL, USA) equipped with an EDS probe (Quantax 75 with XFlash 630H Detector, Bruker Nano GmbH, Berlin, Germany). Dry specimens were mounted on stubs using conductive tape and were analyzed without sputter-coating, using a backscattered electron (BE) detector and an accelerating voltage of 15 kV in a surface-charge reduction mode. This method was used to display the surface morphology of the specimens and, in particular, the distribution of filler particles, their shape, and dimensions.

Three randomly selected fields were acquired for each specimen (500×, 2000×, 5000×) for morphological observation of the surface and with the EDS probe (300 μm × 300 μm fields) in full-frame mode at 150 s acquisition time. The data acquired by EDS were averaged for each specimen and element, the wt% in the ≈1 µm superficial layer was displayed. These data were statistically analyzed to assess significant differences in surface chemical composition among the tested materials. Elemental distribution at the surface level was visually obtained in map mode (5000×) using an acquisition time of 600 s.

### 2.3. Surface Roughness (SR) Analysis

Surface roughness was determined on each disk (*n* = 12) at four randomly selected spots on the surface of each specimen using a profilometric surface contact measurement device (RTP 80-TL90, LTF SpA-Borletti, Antegnate, Italy). A Gaussian filter and a cut-off level of 0.25 were used. A 1.75 mm-long path was measured in one single scan, perpendicular to the expected grinding grooves, using a standard diamond tip (tip radius = 2 µm, tip angle = 90°). The arithmetic mean deviation of the surface roughness profile (Ra), the root mean square average of the profile heights over the evaluation length (Rq), the maximum height of the profile (Rt), and the average maximum height of the profile on five sampling lengths within the evaluation length (RzDIN) were calculated.

### 2.4. Surface Free Energy (SFE) Analysis

**Figure 1:**
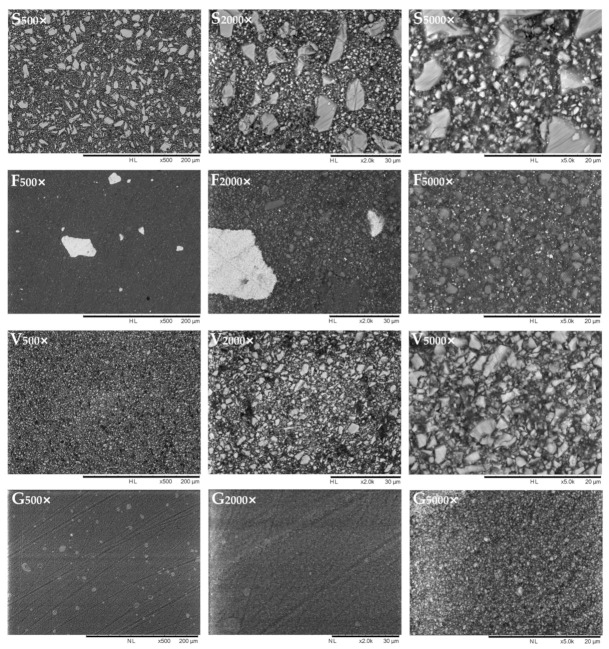
Representative SEM micrographs (500×, 2000× and 5000×) of the tested polymeric materials’ surfaces acquired in backscattered mode. Under such electron detection conditions, elements with relatively high atomic number (Sr, Zr, and especially Ba and Yb) reflect electrons more than elements with lower atomic number (C). In this way, they are depicted as white-ish while the organic resin matrix is black. Elements such as F, Al, and Si provide intermediate gray-scale values. This observation allows to better highlight filler size and shape and provides a preliminary qualitative insight on fillers’ composition.

A 5 μL drop of ultrapure, HPLC-grade water was placed on each of the seven randomly selected specimens for each material (Figure 1). Then, the drop of water was photographed with a reflex camera (EOS-90D equipped with 100 mm macro lens and controlling a Speedlite 600 EX flash with 40 cm bouncer for background illumination, all from Canon, Tokyo, Japan) that was stabilized on a tripod to obtain an orthogonal image. Contact angles were determined using the sessile drop method and a computer-aided contact angle measurement software (Rasband, W.S., ImageJ, U.S. National Institutes of Health, Bethesda, MD, USA). Left and right contact angles were averaged (θ), and the surface free energy (γ_sv_) was calculated according to the formula:

(1) $$\cos\;\theta =-1+2\;\left(\frac{\gamma_{\mathrm{sv}}}{\gamma_{\mathrm{lv}}}\right)$$

considering that the total surface free energy of water (γ_lv_) at the temperature at which the experiments were performed (20 °C) is 72.8 mJ/m^2^.

### 2.5. Microbiological Procedures

Culture media were obtained from Becton-Dickinson (BD Diagnostics-Difco, Franklin Lakes, NJ, USA), and reagents were obtained from Sigma-Aldrich (Sigma-Aldrich, St. Louis, MO, USA). Mitis salivarius bacitracin agar (MSB agar) plates were inoculated with *Streptococcus mutans* (ATCC 35668) and incubated in a 5% CO_2_-supplemented environment at 37 °C for 48 h. A pure culture of the microorganism in the brain-heart infusion broth (BHI) was obtained from these plates after incubation in a 5% CO_2_-supplemented environment at 37 °C for 12 h. Cells were harvested by centrifugation (2200 rpm, 19 °C, 5 min), washed twice with sterile phosphate-buffered saline (PBS), and resuspended in the same buffer. The cell suspension was subsequently subjected to sonication (sonifier model B-150; Branson, Danbury, CT, USA; operating at 7W energy output for 30 s) to disperse bacterial chains. Finally, the suspension was adjusted to 1.0 optical density on the McFarland scale, corresponding to a concentration of approximately 6.0 × 10^8^ cells/mL.

According to a previously published protocol, paraffin-stimulated whole saliva was obtained from five healthy donors [30]. The Institutional Review Board of the University of Milan approved the use of saliva (protocol codename SALTiBO-2017), and written informed consent was obtained from the donors. They refrained from oral hygiene for 24 h, had no active dental disease, and did not use antibiotics for at least three months. Saliva was collected before the beginning of the COVID-19 pandemic, and the donors were not subjected to additional tests regarding their infective status with SARS-CoV-2. Chilled test tubes were used for saliva collection. Saliva was pooled, heated to 60 °C for 30 min and centrifuged (12,000× *g* at 4 °C for 15 min). After that, the supernatant was collected into sterile tubes and stored at −20 °C, to be thawed at 37 °C for 1 h before use.

### 2.6. Bacterial Adherence

Twelve disks for each RBC and curing group were positioned on the bottom of 48-well microplates to evaluate *S. mutans’* adherence to the surfaces of the tested materials. Disks were sterilized using a hydrogen peroxide gas-plasma chemiclave (Sterrad, ASP, Irvine, CA, USA) operating at a low temperature (42 °C) to reduce modifications of the specimen surfaces. Salivary pellicle formation was simulated by covering sterile disks with 300 µL of thawed sterile saliva for 24 h. Then, excess saliva was discarded by aspiration; a total of 500 µL of sterile BHI supplemented with 3 wt% sucrose and 500 µL of *S. mutans* suspension were inoculated into each well. The plates were incubated in a 5% CO_2_-supplemented atmosphere at 37 °C for 2 h. Subsequently, viable biomass adherent to the surface of the disks was assessed as follows.

### 2.7. Viable Biomass Assessment

The viable biomass assessment was performed as previously described [30]. Briefly, two stock solutions were prepared by dissolving 5 mg/mL of 3-(4,5)-dimethylthiazol-2-yl-2,5-diphenyltetrazolium bromide (MTT) and 0.3 mg/mL of N-methylphenazonium methyl sulphate (PMS) in sterile PBS. The solutions were stored at 2 °C in light-proof vials until the day of the experiment when a test solution (TS) was prepared by mixing MTT stock solution, PMS stock solution, and sterile PBS in a 1:1:8 ratio. A lysing solution (LS) was prepared by dissolving 10 vol% sodium dodecyl sulfate and 50 vol% dimethylformamide in distilled water. TS and LS were brought to 37 °C before use. After 2 h of incubation, disks were transferred into new 48-well plates containing 300 µL of TS in each well.

The plates were incubated at 37 °C under light-proof conditions. During incubation, electron transport across the microbial plasma membrane and, to a lesser extent, microbial redox systems converted the yellow salt to insoluble purple formazan crystals. The conversion at the cell membrane level was facilitated by the intermediate electron acceptor (PMS). After one hour, the TS solution was carefully removed, and 300 µL of LS was added to each well. The plates were then stored under light-proof conditions for one additional hour (room temperature) to allow dispersion of the formazan crystals into the surrounding solution. Subsequently, 100 µL of the supernatant was transferred to a 96-well plate, and the absorbance was measured at a wavelength of 550 nm using a spectrophotometer (Genesys 10-S, Thermo Spectronic, Rochester, NY, USA). Results were expressed as relative absorbance in optical density (OD) units corresponding to adherent, viable, and metabolically active biomass.

### 2.8. Bioreactor Procedures

Biofilm formation was simulated under continuous flow conditions using a modified commercially available drip-flow bioreactor (MDFR; DFR 110, BioSurface Technologies, Bozeman, MT, USA). The modified design allowed the placement of customized polytetrafluoroethylene (PTFE) trays containing 27 holes, in which each specimen was tightly fixed on the bottom of the flow cell, exposing its surface to the surrounding medium. Before the experiments, all tubing and specimen-containing trays of the MDFR were sterilized using the chemiclave (Sterrad). The whole apparatus was then assembled inside a sterile hood [31].

A total of 10 mL of thawed sterile saliva was placed into each flow cell to allow the formation of a salivary pellicle on the surface of the tested disks (n = 12 for each material and curing group). Then, the MDFR was incubated at 37 °C for 24 h. After incubation, saliva was removed by gentle aspiration. A total of 10 mL of the previously prepared *S. mutans* suspension was then placed into each flow cell, and the MDFR was incubated at 37 °C for 4 h to allow bacterial adherence. Then, a constant flow of sterile modified artificial saliva medium [31] including 2.5 g/L mucin (type II, porcine gastric), 2.0 g/L bacteriological peptone, 2.0 g/L tryptone, 1.0 g/L yeast extract, 0.35 g/L NaCl, 0.2 g/L KCl, 0.2 g/L CaCl_2_, 0.1 g/L cysteine HCl, 0.001 g/L hemin, and 0.0002 g/L vitamin K_1_ was provided by a peristaltic pump at a flow rate of 9.6 mL/h. The MDFR was operated for 24 h to allow the development of a multilayer biofilm on the specimens’ surfaces. At the end of the incubation, the flow was stopped, and the trays were extracted from the flow cells. The specimens were carefully removed from the trays using a pair of sterile tweezers and gently rinsed with sterile phosphate-buffered saline (PBS) at 37 °C to remove non-adherent cells. The specimens were then placed into sterile 48-well plates, and the adherent, viable biomass was assessed as previously specified.

### 2.9. Statistical Analysis

Statistical analyses were performed using the JMP 10.0 software (SAS Institute, Cary, NC, USA). Normal distribution of data was checked using the Shapiro–Wilk test, and homogeneity of variances was verified using Levène’s test. Means, standard deviations, and standard errors were calculated from the raw data. A two-way analysis of variance (ANOVA) was used to analyze the surface roughness, SFE, EDS, and biomass datasets, considering the material type and the curing time as fixed factors. Tukey’s HSD test was employed for post-hoc analysis (*p* < 0.05).

## 3. Results

### 3.1. Surface Characterization

**Figure 2:**
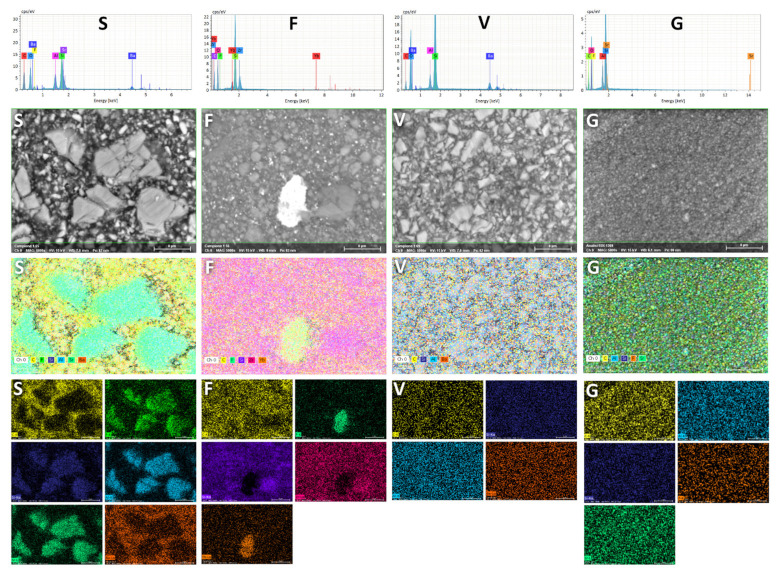
EDS analysis. For each tested polymeric material, from top to bottom are shown a typical spectrum of the surface showing elemental detection, an SEM backscattered micrograph at 5000×, the superimposed false-color image showing elemental detection and smaller pictures depicting each acquired channel. It can be clearly seen that the fillers of the SDR material (**S**) appear very similar in shape, dimension and composition to a resin-based glass ionomer material (F-Al-Si-Sr glass) to which micronized barium glass was added. Filtek Bulk Fill (**F**) shows nanoparticles and clusters of YbF_3_ embedded in silica and zirconia nanoparticles and nanoclusters. The composition of Admira Fusion X-tra (**V**) and Universal Flo (**G**) is extremely homogeneous, with the first belonging to the micro-hybrid resin composite class (microfillers+nanofillers) while the second one is nanofilled.

SEM-EDS observation showed that SDR surfaces exposed filler with different dimensions, in the range of 5–10 µm (macro), about 1 µm (micro), as well as nanofillers (Figure 1). EDS mapping (Figure 2) partly confirmed the manufacturer’s specification, identifying macro fillers fabricated of fluoro aluminosilicate glass with Sr and smaller filler particles in the range of about 1µm fabricated by barium glass. This material showed a filler shape and composition similar to that of a resin-modified glass-ionomer [32], to which micronized Ba glass was added. Filtek Bulk Fill exposed zirconia and silica nanofillers and nanoclusters, as expected. Interestingly, both clusters and finely dispersed nanoparticles of the ionic compound YbF_3_ were identifiable, having an inhomogeneous distribution. Due to the high atomic number of ytterbium, such particles were highly electron-reflective in backscattered mode and easily identifiable. The other two materials showed a very homogeneous filler distribution. Admira Fusion X-tra displayed barium aluminosilicate glass particles in the 1–5 µm range (microhybrid), whereas Universal Flo showed sub-micron filler particles made of Si, Al, Sr, and F (nanofilled).

Considering the control material, Al presence was found that can be related to strontium glass. Indeed, strontium cannot stand alone, and strontium oxide needs to be included as a network-modifier in a SiO_2_-Al_2_O_3_ glass, which is why the Al signal was detected [33]. Fluoride was contained in the control material as the ionic compound LaF_3_; yet, the lanthanum signal was not identified, likely being below the instrument’s detection limit.

The quantitative analysis of the tested materials’ surface is shown in Table 2. The statistical analysis showed that the curing time did not influence the materials’ surface elemental composition, and no significant interaction was demonstrated between the tested factors. Therefore, results were grouped by the material.

**Figure 3:**
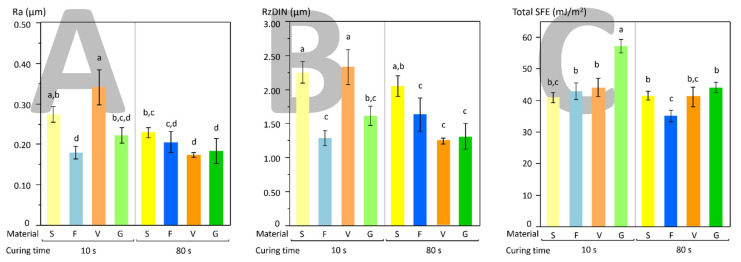
Graphs depicting the results of surface characterization in terms of surface roughness (**A**) Ra parameter and (**B**) RzDIN parameter and surface free energy (**C**). Curing time had a huge impact on both surface roughness and SFE, with differences between tested polymeric materials being significantly reduced after extended curing (80 s). Different superscript letters indicate significant differences between materials (Tukey’s test, *p* < 0.05) for a given element.

Surface roughness datasets were not normally distributed; therefore, each dataset was log-transformed before statistical analysis to approach a normal distribution. Material and curing time significantly influenced all surface roughness parameters calculated in the present study (*p* < 0.001). A significant interaction was highlighted between the considered factors on all parameters. Rq and Ra showed a similar trend. After 10 s curing time, V showed significantly higher Ra values compared with G and F, and S showed higher Ra compared with F (Figure 3A). After 80 s, S showed significantly higher Ra than V and G. Then again, all materials showed very similar Ra values after 80 s curing, around 0.2 µm. Rt and Rz showed a similar trend. After 10 s of curing time, S and V showed significantly higher Rz values than F and G (Figure 3B). After 80 s, S showed significantly higher Rz values compared with all other materials.

Surface free energy assessment (Figure 3C) showed that G (control) had a significantly higher SFE than the tested bulk-fill materials after 10 s of curing time. After 80 s of curing, both G and S showed significantly higher SFE than F, but differences were less pronounced than after 10 s of curing.

### 3.2. Microbiological Evaluation

**Figure 4:**
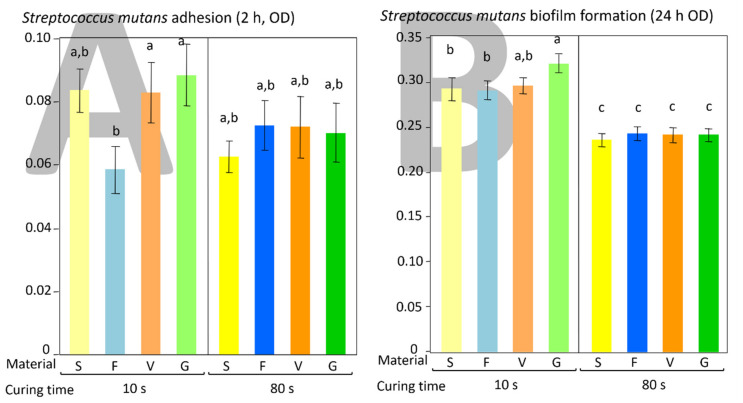
Results of the microbiological behavior of the tested materials in terms of bacterial adherence (**A**) and biofilm formation (**B**) by *S. mutans*. A highly significant decrease in both adherence and biofilm formation can be observed on materials cured for an extended time (80 s). Interestingly, differences in adherence and biofilm formation between materials disappeared after extended curing time (80 s). Different superscript letters indicate significant differences between materials (Tukey’s test, *p* < 0.05) for a given element.

No significant influence of the considered factors on bacterial adherence was highlighted. The post-hoc test showed significantly lower F surfaces adherence than V and G (*p* = 0.044 and *p* = 0.0265, respectively, Figure 4A).

Curing time significantly influenced biofilm formation (*p* < 0.0001), while no significant influence of the material, and no significant interaction between the considered factors were detected (*p* = 0.0772, and *p* = 0.341, respectively). A post-hoc test showed significantly higher biofilm formation on G (control) surfaces compared with S and F (*p* = 0.0236 and *p* = 0.0299, respectively, Figure 4B).

## 4. Discussion

The mutual interactions between the surfaces of polymeric dental materials and overlying biofilms are complex and far from being fully understood. The conventional wisdom is that surface roughness is the main parameter influencing microbial adherence and biofilm formation, with other parameters, such as surface free energy and chemical composition playing a minor role [21]. The purpose of this study was to evaluate the influence of light-curing time on the adherence and biofilm formation by *Streptococcus mutans* on three bulk-fill composites and to put into relation such data with the surface characteristics. The first null hypothesis that there is no difference in bacterial adherence or biofilm formation between the tested polymeric materials must be rejected in parts. Indeed, after 10 s of polymerization time, significant differences were found between materials both for bacterial adherence and biofilm formation, whereas no significant differences were found after 80 s curing. The second null hypothesis must be entirely rejected since all tested materials showed lower biofilm formation after an extended curing time (80 s).

Our results showed that a reduced curing time produced higher surface roughness on SDR and Admira Fusion X-tra, while the extended curing time generally produced similar lower values. Surface roughness is considered a crucial parameter in influencing all phases of microbial colonization [34,35]. High surface roughness values are believed to improve microbial adherence by providing attachment sites and reducing the shear force of the flow on bacterial cells and microcolonies [36]. However, the present study showed a poor correlation between roughness data and microbiological behavior of materials, both in terms of bacterial adherence and biofilm formation. In particular, adherence and biofilm formation were similar for all tested materials when cured for an extended time (80 s), and the only factor influencing the microbiological behavior was the curing time.

There is, unfortunately, no consensus on the influence of surface roughness on the microbiological behavior of resin-based materials in the literature. The same issue can be found when considering bulk-fill materials. Two studies suggest that surface roughness significantly affects bacterial adherence and biofilm formation [27,37], while most show no effect of this parameter on microbial colonization [13,25,26,28,38]. In particular, Somacal et al. in 2020 evaluated the effect of pH cycling and simulated toothbrushing on the surface roughness and 24 h-biofilm formation (not adherence) of some bulk-fill materials [25]. One of the tested materials (Filtek Bulk Fill) was also tested in the present study. They did not find any correlation between roughness values and biofilm formation, agreeing with the present study’s data. In the same year, Park et al. studied the influence of surface roughness on microbial adherence after applying finishing procedures to some polymeric materials, among which was a bulk fill [26]. They only found a weak correlation between surface roughness and *S. mutans* adherence to the specimens. Bilgili et al. evaluated *Streptococcus mutans* and *Streptococcus mitis* 24 h-biofilm formation (not adherence) to bulk-fill resin composites in relation to their surface characteristics [13]. In particular, they evaluated two of the materials tested in the present study (Filtek Bulk Fill and Admira Fusion X-tra). They concluded that the surface roughness did not affect biofilm formation. Cazzaniga et al. evaluated the influence of surface roughness of microhybrid, nanohybrid, nanofill, and bulk-fill composites finished with several finishing/polishing systems on *S. mutans* biofilm formation [28]. The polishing systems significantly influenced surface roughness, yet surface roughness was not found to influence biofilm formation. Several other studies demonstrated no correlation between surface roughness and *S. mutans* colonization of polymeric surfaces [29,30,39].

On the other hand, Soliman et al. in 2019 evaluated the influence of surface roughness on *S. mutans* adherence to bulk-fill materials treated using different polishing systems [37]. One of the materials, Filtek Bulk Fill, was also tested in the present study. Contrarily to our results, they found a significant association between surface roughness and bacterial adherence to the tested surfaces. One of the surface treatments in Soliman’s experiment was curing the materials against a mylar strip, which is acknowledged to produce a smooth surface, similar to the protocol adopted in the present study, that involved curing against glass plates.

It is known that other characteristics such as surface free energy can significantly influence bacterial adherence both in vivo and in vitro [21,36]. This influence is reduced over time as the biofilm formation phase progresses [40]. However, previous studies found no significant relationship between the hydrophobicity of polymer-based composites and bacterial adherence [29,41,42,43]. Our results showed that the control material (Universal Flo) displayed a significantly higher SFE than the tested bulk-fill materials when light-cured for 10 s, while these differences were much less pronounced after 80 s. Microbiological data of biofilm formation showed a similar trend, with the control material showing the highest biofilm development when light-cured for 10 s, while no differences between groups were seen after 80 s of curing time. While it is generally accepted that higher surface free energy values correlate with higher *S. mutans* adherence [29], only Bilgili et al. [13] evaluated the influence of this parameter on the microbiological performances of bulk-fill surfaces. They tested four different bulk-fill materials and found no significant influence of SFE on *S. mutans* biofilm formation (24 h). Such outcome agrees with the present study results, where a higher SFE, correlating with higher biofilm formation, was only found for the control material.

These experimental findings suggest that biofilm formation is mainly influenced by the surface chemical composition of the material, including filler size, shape, distribution, and matrix composition. The surface microanalysis and elemental composition (SEM-EDS) provided data on the composition of the external ≈1 μm layer of the tested materials. A previous study [43] on several conventional RBCs showed that their filler to resin matrix ratio could influence biofilm formation. Indeed, a higher amount of inorganic filler presence on the surface is related to reduced bacterial colonization. SDR showed the lowest filler presence in the present study, whereas Admira Fusion X-tra showed the highest. However, this characteristic did not influence bacterial adherence or biofilm formation. However, it must be noted that the tested bulk-fill materials generally have much lower filler content than conventional RBCs. This feature, along with filler shape and dimensions, is usually selected for bulk-fill composition to improve the depth of cure of the polymeric materials [3,4,7]. In the present study, a nanofill flow composite was used as a control since it had characteristics, such as a filler/matrix ratio similar to the tested bulk-fill materials. Therefore, a relatively low filler to resin matrix ratio may explain the lack of correlation between this characteristic and the microbiological performance of the tested materials.

Furthermore, it was found that SDR, Filtek Bulk Fill, and Universal Flo contained fluoride in their composition, as fluoro aluminosilicate glass, YbF_3_, or LaF_3_, respectively. Nevertheless, despite its proven antimicrobial and bacteriostatic effect even at low concentrations, no influence of fluoride on microbial adherence or biofilm formation was found. A possible explanation may be that fluoride is firmly incorporated into the material and resin matrix without expressing significant release once polymerized. Literature data on other fluoride-containing conventional and bulk-fill polymeric materials tested under similar biofilm formation conditions, including forming a salivary pellicle, agrees with the present findings [28,30]. Further studies may evaluate the fluoride release capacity of such materials under biofilm formation conditions.

Finally, other factors can contribute to bacterial adherence, such as the resin matrix composition [15,44] and the amount of leaching of residual unpolymerized monomers [45]. In the present study, all materials were extensively rinsed using a standard protocol [31,43] to minimize the impact of possible unpolymerized monomer release or fluoride burst on the microbiological behavior. In agreement with the present study’s data, progressively reduced biofilm formation was found on the surfaces of conventional RBCs with increasing curing time [24]. This behavior was explained by increasing the degree of conversion at the RBC surface and decreasing the amount of leachates. Despite incorporating different and supposedly more efficient photoinitiator systems in bulk-fill polymeric materials [9,12], they seem to behave similarly to their conventional counterparts, at least from the point of view of the influence of the curing time on their microbiological behavior. For instance, Alshali et al. [46] showed that elution of residual monomers from SDR did not differ from conventional resin composites. Then again, bacteria and especially *S. mutans* can be deeply influenced by urethane-containing monomers, notably UDMA. With urethane-based derivatives, the latter is currently being used to replace BisGMA to avoid drawbacks such as high viscosity, toxicity, and estrogen-like effects on the human body. Kim et al. in a very recent paper [47], demonstrated that UDMA could contribute to the development of secondary caries around UDMA-containing polymeric materials by prompting *S. mutans* biofilm formation, enhancing its oxidative tolerance, and enabling it to shift its carbon flow toward the ATP generation required for persistence and cariogenicity. The tested materials all contained UDMA or its derivatives, including Admira Fusion X-tra based on ORMOCER technology incorporating multifunctional urethane and thioether (-meth) acrylate alkoxysilanes as sol-gel precursors [48]. This consideration may explain why all materials had similar microbiological behavior after curing for 80 s. In addition to that, Filtek Bulk Fill was the only material that did not contain TEGDMA in its composition. Together with an improved photoinitiator system, this consideration may explain its significantly lower microbial adherence after only 10 s of curing.

Some choices were made for the experimental design of the present study based on a reductionistic approach. Only 10 s and 80 s of light-curing time were tested, which may be too low or exceed the curing times suggested by the manufacturers, ranging from 20 s (SDR Surefil, Admira Fusion X-tra, Universal Flo) to 40 s (Filtek Bulk Fill). Based on the results of a previous study performed with conventional composites [24], the time categories of the present study were chosen to see best if the microbiological behavior of the tested materials was influenced by the curing time similarly to the conventional composites, despite differences in composition and photoinitiators. Furthermore, *S. mutans* monospecies biofilm is an oversimplistic microbiological model compared with a fully-grown artificial oral microcosm. The bacterium, however, allows the development of a cariogenic biofilm resembling its’ in vivo counterparts, thus providing the best comparability of the gathered results with the literature. Future studies on this topic should include artificial oral microcosms based on bioreactor-grown mixed plaque inocula [49].

## 5. Conclusions

Like conventional RBCs, prolonged curing time (80 s) reduced bacterial adherence and biofilm formation on all tested bulk-fill polymeric materials. Improved photoinitiator systems, providing optimally cured materials after 80 s, and resin matrix composition (UDMA promoting adherence and biofilm formation) may explain these results.

Surprisingly, when bulk-fill composites were extensively cured, no difference in bacterial adherence or biofilm formation could be seen comparing the different materials. Furthermore, no correlation between surface characteristics (surface roughness, surface free energy, elemental composition, fluoride presence, filler/resin ratio) and microbiological data could explain such behavior. Compared with conventional RBCs where surface characteristics, especially surface chemistry, influences microbiological behavior, the different performance of bulk-fill polymeric materials may have important implications in secondary caries occurrence and restoration longevity. Comparative clinical studies are needed in the long term to assess this possibility.

## Figures and Tables

**Table 1:** Codename, manufacturer, and composition of the resin-based materials tested in the present study.

Codename	Material	Manufacturer	Organic Matrix	Filler (wt%, vol%)
S	SDR Surefil	Dentsply Sirona, York, PA, USA	UDMA, TEGDMA, EBPDMA	Ba-Al-F-Si glass, Sr-Al-F-Si glass 68 wt%, 45 vol%
F	Filtek Bulk Fill Posterior	3M, St Paul, MN, USA	AUDMA, UDMA, DDMA	Nanofillers and clusters of SiO_2_; ZrO; YbF_3_ 64.5 wt%, 50.4 vol%
V	Admira Fusion X-tra	VOCO GmbH, Cuxhaven, Germany	ORMOCER	Ba-Al-Si glass; SiO_2_ 84 wt%, 65 vol%
G (Control)	G-aenial Universal Flow	GC Corp. Europe, Leuven, Belgium	UDMA, TEGDMA, Co-monomer dimethacrylates.	SiO_2_ (16 nm), Sr glass (200 nm), LaF_3_ 69 wt%, 50 vol%

Abbreviations: Bis-GMA: bisphenol A glycol dimethacrylate; ethoxylated bis-GMA: EBPDMA; TEGDMA: triethylene glycol dimethacrylate; UDMA: urethane dimethacrylate; aromatic urethane dimethacrylate: AUDMA; 1, 12 dodecane-DMA: DDMA; urethane-based organically modified silicic acid: ORMOCER.

**Table 2:** Energy-dispersive X-ray spectroscopy (EDS) compositional analysis of specimens’ surface layer after finishing and before microbiological challenges. Means (±1 SD) are displayed. Inorganic fraction is depicted as the sum of all elements constituting the fillers, as opposed to the organic matrix evidenced by the carbon content. Two-way ANOVA showed that material, not curing time factor, was highly significant (*p* < 0.0001); therefore, results are provided as the average composition for each material. Different superscript letters indicate significant differences between materials (Tukey’s test, *p* < 0.05) for a given element.

wt%	S	F	V	G (Control)
C	40.05 (2.74) ^a^	33.34 (3.92) ^b^	22.59 (4.51) ^c^	24.72 (3.29) ^c^
O	30.20 (0.62) ^c^	32.34 (1.43) ^b^	39.82 (1.65) ^a^	41.11 (0.89) ^a^
F	3.81 (0.66) ^a^	1.39 (0.27) ^b^	0.00 (0.00) ^c^	0.99 (0.19) ^b^
Al	4.23 (0.25) ^a^	0.00 (0.00) ^c^	3.10 (0.34) ^b^	4.47 (0.30) ^a^
Si	10.33 (0.42) ^c^	18.66 (1.36) ^b^	22.76 (1.99) ^a^	18.22 (1.43) ^b^
Sr	5.01 (0.56) ^b^	0.00 (0.00) ^c^	0.00 (0.00) ^c^	10.49 (0.69) ^a^
Zr	0.00 (0.00) ^b^	10.80 (1.35) ^a^	0.00 (0.00) ^b^	0.00 (0.00) ^b^
Ba	6.37 (1.04) ^b^	0.00 (0.00) ^c^	11.78 (1.14) ^a^	0.00 (0.00) ^c^
Yb	0.00 (0.00) ^b^	3.47 (0.38) ^a^	0.00 (0.00) ^b^	0.00 (0.00) ^b^
Inorganic fraction	29.75 (2.39) ^b^	34.33 (2.51) ^a^	37.64 (3.36) ^a^	34.18 (2.41) ^a,b^

## Data Availability

The data presented in this study are either present in the article body or available upon request from the corresponding author.
